# Analysis of incidence and prognostic factors for ipsilateral breast tumour recurrence and its impact on disease-specific survival of women with node-negative breast cancer: a prospective cohort study

**DOI:** 10.1186/bcr1531

**Published:** 2006-07-19

**Authors:** Michelle K Nottage, Karen A Kopciuk, Anjela Tzontcheva, Irene L Andrulis, Shelley B Bull, Martin E Blackstein

**Affiliations:** 1 Royal Brisbane Hospital, Herston Road, Herston, Queensland 4029, Australia; 2 Division of Population Health and Information, Alberta Cancer Board, 1331-29th Street N.W., Calgary, AB, T2N 4N2, Canada; 3 Department of Mathematics and Statistics, University of Calgary, 2500 University Drive NW, Calgary, AB, T2N 1N4, Canada; 4 Samuel Lunenfeld Research Institute, Prosserman Centre For Health Research, Mount Sinai Hospital, 60 Murray Street, Box 18, Toronto, ON, M5T 3L9, Canada; 5 Department of Public Health Sciences, University of Toronto, 155 College Street Toronto, ON, M5T 3M7, Canada; 6 Department of Molecular and Medical Genetics, University of Toronto, 1 King's College Circle, Toronto, ON, M5S 1A8, Canada; 7 Mount Sinai Hospital, 600 University Avenue, Toronto, ON, M5G 1X5, Canada

## Abstract

**Introduction:**

This study had three aims: to establish the incidence of ipsilateral breast tumour recurrence (IBTR) in a community treatment setting, to evaluate known factors – in particular younger age (< 40 years) – predictive for local recurrence, and to assess the impact of local recurrence on disease-specific survival (DSS).

**Methods:**

A consecutive series of 1,540 women with node-negative breast cancer, diagnosed between the ages of 18–75 years, were prospectively accrued between September 1987 and September 1999. All had undergone a resection of the primary breast cancer with clear margins, an axillary lymph node dissection with a minimum of four sampled nodes, and breast-conserving surgery (of any type).

**Results:**

During the study follow-up period, 98 (6.4%) IBTRs and 117 (7.6%) deaths from or with breast cancer were observed. The median time to IBTR was 3.1 years and to death from or with disease was 4.3 years. In the multivariate Cox proportional hazards (PH) regression model for IBTR with adjuvant therapy factors, independent risk factors included age < 40 years (relative risk (RR) = 1.89, 95% confidence interval (CI) of 1.00 – 3.58), presence of intraductal disease (RR = 1.81, 95% CI = 1.15–2.85) and histological grade ('G2' or G3 versus G1: RR = 1.59, 95% CI = 0.87–2.94). In the multivariate Cox PH regression model for DSS with adjuvant therapy factors, independent risk factors included previous IBTR (RR = 2.58, 95% CI = 1.41–4.72), tumor size (1–2 cm versus < 1 cm: RR = 1.95, 95% CI = 1.05–3.64, > 2 cm versus < 1 cm: RR = 2.94, 95% CI = 1.56–5.56), progesterone receptor status (negative or equivocal versus positive or unknown: RR = 2.15, 95% CI = 1.36–3.39), lymphatic invasion (RR = 1.78, 95% CI = 1.17–2.72), and histological grade ('G2' or G3 versus G1: RR = 8.59, 95% CI = 2.09–35.36). The effects of competing risks could be ignored.

**Conclusion:**

The Cox PH analyses confirmed the importance of known risk factors for IBTR and DSS in a community treatment setting. This study also revealed that the early occurrence of an IBTR is associated with a relatively poor five-year survival rate.

## Introduction

Breast-conserving surgery (BCS) has been a standard of care for some considerable time [1,2]. Local recurrence after mastectomy is associated with low 5-year survival rates of 22 to 40% [3-5]. However, the relationship between local recurrence after BCS and metastatic disease or survival is more complex. Local recurrence in these women is often amenable to salvage therapy (that is, mastectomy or further BCS), and early randomized trials of BCS versus mastectomy, or BCS with and without radiation, demonstrated that survival is comparable despite rates of local recurrence in women treated with BCS alone of 25% [6-8] to 35% [9]. Further analysis of the pivotal National Surgical Adjuvant Breast and Bowel Project B06 trial showed that local recurrence was a strong predictor of reduced survival, but it seemed to act as a marker for unfavourable disease rather than as a source for the development of metastases [10]. This trial reported that women experiencing local recurrence had a relative risk (RR) for developing distant disease 3.41-fold that of women free of local recurrence.

In comparison with clinical trial participants, community-based observational patient cohorts may provide enhanced generalizability. Beginning in 1987, we have followed a consecutive series of 1,540 women with node-negative breast cancer treated at eight Toronto hospitals [11]. Women in this series were treated in accordance with the community standards of the time. Because, overall, women with node-negative disease have favourable prognoses, analysis of local recurrence is more straightforward than in node-positive women, in whom high rates of metastatic disease present a strong competing risk. With a minimum of 2.3 years of follow-up, we used this large prospectively accrued cohort to estimate the incidence of local recurrence and determine its effect on disease-specific survival (DSS). As a secondary question, we investigated the effect of patient age on these outcomes.

## Materials and methods

### Patient eligibility, measured features and clinical follow-up

Women aged 18 to 75 years with node-negative breast cancer were accrued from September 1987 until September 1999. To be eligible for this prospective study, women had to have undergone a resection of a primary breast cancer with clear margins, and an axillary lymph node dissection with a minimum of four sampled nodes, based on pathology review. The median number of nodes removed was 11; the number of nodes removed ranged from 4 to 38 nodes. All patients with T2 tumours underwent staging with the use of bone scan, abdominal ultrasound/abdominal computed tomographic scan and chest X-ray. Eligible women were invited to participate by their treating physician. Written informed consent was obtained and final eligibility was based on chart review. Exclusion criteria included inadequate assessment of histological grade, inadequate staging, synchronous breast primary tumours, surgeon or patient refusal, or previous malignancies. For the purposes of this present report we included only women who had undergone BCS (of any type) and had any follow-up information.

Patient and pathological features recorded, as reported previously [11], included age, menopausal status, tumour size and grade, endothelial space invasion, multicentricity (two or more separate (usually) invasive primary breast cancers), hormone receptor status, and presence of *in situ *disease.

Treatment and subsequent follow-up data were collected in a standardized format. Charts were reviewed every 3 months during the first 2 years after diagnosis, every 6 months until 5 years after diagnosis, and every year thereafter. Follow-up for this report was terminated as of 10 January 2002, with an additional 6 months allowed for verification purposes. The time interval from the median participant entering the study to the termination date for analysis was 10.9 years. Follow-up data collected for each patient included treatment received, local and distant recurrences, and cause of death, with the corresponding dates. Local and distant recurrences were confirmed by review of reports or imaging conducted by MEB.

### Statistical analyses

A total of 1,540 women met the eligibility criteria for this report. Descriptive statistics compared the frequency distribution of measured patient and pathological features between groups defined by age (age < 40 years versus age ≥ 40 years).

### Ipsilateral breast tumour recurrence and disease-specific survival

The two outcomes of interest in this study are time to local recurrence (ipsilateral breast tumour recurrence; IBTR) and time to death with or from disease. Both invasive and intraductal IBTRs were included as events for the analysis of local recurrence. Deaths from and with disease defined events for DSS. Only prognostic factors previously identified in the literature were evaluated in the statistical analyses. Univariate survival analysis of each measured prognostic factor was conducted via the log-rank test with Kaplan–Meier curves and via the likelihood ratio statistic for Cox proportional hazards (PH) models [12-14].

Patients currently lost to follow-up (*n *= 26) were censored at their last known follow-up time for either event of interest. Censoring for IBTR could also occur for three reasons. IBTR-free women were censored at their death date (death from any cause) or at the study termination date. In addition, women who experienced a distant metastasis within 12 weeks before a diagnosis of IBTR (*n *= 10) were censored for IBTR at their diagnosis date for the distant metastasis [15]. This time frame includes only women with an isolated IBTR and eliminates those who had a synchronous systemic recurrence because this might lead to a secondary finding of an IBTR. Deaths from or with breast cancer were included in the analysis of DSS, whereas women who died for other reasons (*n *= 80) were censored at their death times.

Using a Cox PH model, statistically significant (*p *≤ 0.05) or borderline significant (*p *< 0.1) prognostic factors identified from the univariate survival analyses, together with factors selected for hypothesis testing, were evaluated in multivariable survival models. Because any form of treatment (radiotherapy, hormonal or chemotherapy) can be a confounding factor with patient and pathological features, all forms of treatment were forced into the final model. To evaluate the hypothesis that younger women (age < 40 years) experienced a higher rate of IBTR than older women (age ≥ 40 years), this factor was included in the multivariate modelling process for IBTR outcome as well. Pairwise interactions between the factor for young age and each of the other factors associated with IBTR were assessed separately in bivariate models that also included both variables. However, because very few younger women (age < 40 years) experienced a local recurrence (12 of 98), most interactions could not be evaluated because of inadequate power (results not shown).

The effect of time dependence of an IBTR on survival was taken into account in the statistical analyses of DSS by modelling IBTR as a time-dependent covariate in the multivariable Cox PH models. In addition, the landmark method [16] was used to estimate survival after an IBTR; the landmark time of 1.7 years since surgery was chosen because half of the 14 women who had both outcomes had experienced an IBTR by this time. However, by 1.7 years from surgery, 20 women without IBTR were excluded from the estimation of the DSS curves on the basis of IBTR status because some had already died from or with disease (*n *= 8) or were censored (*n *= 12).

### Model assessment and competing risks

The proportional hazards assumption was assessed graphically and formally with a trend test statistic [17]. Scaled Schoenfeld residuals were plotted against survival time for each factor separately and for all variables combined in the multivariate model. A Loess smoothing curve was used for visually detecting trends over time. A formal global test of proportionality for all factors included in a model was based on a χ^2 ^distribution for testing for a trend between the scaled Schoenfeld residuals and survival time.

In this study, women could experience one or both of the events of interest during follow-up: an IBTR, and death from or with breast cancer. If a woman does experience an IBTR, her risk for dying can be altered. Similarly, if a woman experiences a distant recurrence and subsequently dies from or with breast cancer, she is not really at risk for IBTR and so should not be treated as simply censored in any time-to-IBTR analyses. The competing risks between developing an IBTR and dying from or with disease were evaluated by modelling the cumulative incidence function for each event of interest [14] and fitting the proportional subdistribution hazards regression models [18]. The first approach estimates the probability of the event of interest when the patient is subject to the other event without adjusting for the effect of covariates, whereas the second approach directly assesses the effects of covariates on the subdistribution of an event of interest in a competing risks setting. To assess the potential impact of these competing risks on our analyses, we compared estimates of the survivor function and regression coefficients calculated ignoring or explicitly modelling the competing risk.

All analyses were conducted with SAS/STAT^® ^software (Version 8.2) SAS System for Unix [19] and R software (version 2.1) [20].

## Results

### Patient, tumour, and adjuvant treatment characteristics

Nearly two-thirds of the 1,540 eligible patients were postmenopausal at diagnosis (Table 1), and most (91.7%) were 40 years of age or older (mean 56.1 years, SD 11.3 years). Tumours were generally small, with 66% being 2 cm or less. The younger women tended to have larger tumours (larger than 2 cm), to be negative or equivocal for hormone receptor status, and to have grade 3 (G3) tumours.

Most but not all of the patients received radiotherapy. The treatment field was nearly always only the breast area (98%). Just under 50% received adjuvant hormonal therapy and 15.5% received adjuvant chemotherapy; the proportion receiving adjuvant systemic therapy increased over time. Tamoxifen was the sole hormone therapy for 741 of the 758 women who received this adjuvant therapy. The predominant (196 of 239) chemotherapy regimen was CMF (cyclophosphamide–methotrexate–5-fluorouracil). The type of systemic adjuvant therapy differed with age, with younger women receiving less hormonal therapy (18% versus 52%) and more chemotherapy (43% versus 13%) than the older women. Younger women who were oestrogen receptor-positive were about half as likely to receive hormone therapy as older women who were oestrogen receptor-positive (31% versus 57%; data not shown).

### Incidence of IBTR

**Figure 1:**
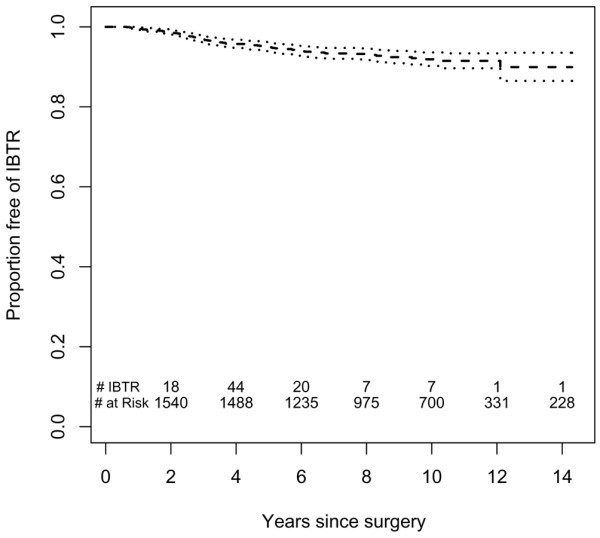
Kaplan–Meier estimate of ipsilateral breast tumour recurrence (IBTR).

**Figure 2:**
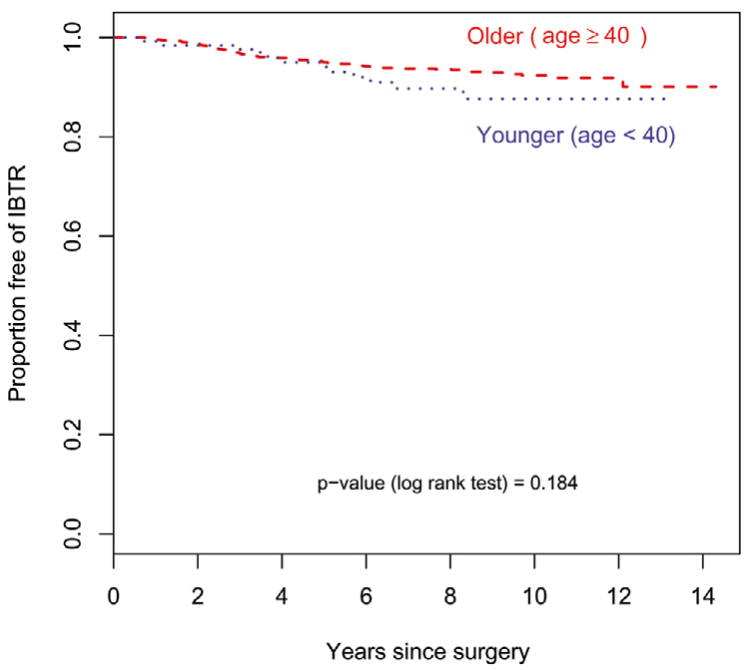
Kaplan–Meier estimate of IBTR stratified by age under or over 40 years. IBTR, ipsilateral breast tumour recurrence.

The incidence of an isolated local recurrence was 6% (*n *= 98) in this community-based study. The median time to IBTR was 3.1 years (SD 2.4), with nearly two-thirds (62 of 98) occurring within 4 years from surgery (Figure 1). Of the 98 IBTRs, 27 were classified as intraductal and 71 as invasive. In the Kaplan–Meier estimate of the time to IBTR stratified according to age at diagnosis (age < 40 years versus age ≥ 40 years), the two curves begin to separate at about 5 years after surgery (Figure 2). The percentage free of IBTR at 8.3 years was 88% for younger women (age < 40 years), in comparison with 93% for older women (age ≥ 40 years), which, however, is not significant based on a simple log-rank test statistic (*p *= 0.47).

### Prognostic factors for local recurrence

Factors associated with the risk of IBTR in univariate analysis in this series (Table 2) included multicentricity and presence of ductal carcinoma *in situ*. The apparent association between histological grade not done or unknown and increased incidence of IBTR is unexplained but may be related to different pathology practices at the treating centres and unknown case-mix variables, as the percentage of times that a histological grade was not done or was unknown varied from 8.04% to 33.62% across the eight hospitals. As expected, women who received radiotherapy experienced fewer local recurrences. Hormone therapy and chemotherapy were also apparently protective against IBTR but to a smaller extent.

Although the age < 40 years variable was not statistically significant in the univariate Cox PH model, to assess the hypothesis about the increased incidence of local recurrence in this age group, this variable was also evaluated in a multivariate model adjusting for the effects of other relevant factors. In a multivariate model, age at diagnosis < 40 years and presence of intraductal disease were found to be independently associated with IBTR, whereas adjuvant therapy (particularly radiotherapy) was associated with reduced risk (Table 2). Histological grade was also significant (likelihood ratio statistic = 7.2 with 2 degrees of freedom, *p *= 0.027), but this was apparently due to the association between IBTR and unknown histological grade. The variable multicentricity lost significance in the multivariate model, but because 11 of 12 women who experienced an IBTR and had areas of multicentricity were also classified as having ductal carcinoma *in situ*, the separate effects of these variables on IBTR could not be evaluated in these data.

### Impact of IBTR on disease-specific survival

The diagnosis of an isolated IBTR significantly affected DSS. In a univariate Cox PH model for DSS, IBTR had a RR of 3.04 (95% confidence interval (CI) = 1.72 to 5.35). This risk diminished only slightly (RR = 2.58, 95% CI = 1.41 to 4.72) in a multivariable Cox PH model for DSS. In addition, we found that an early IBTR diagnosis (within 1.7 years of surgery) resulted in a very poor 5-year survival rate.

**Figure 3:**
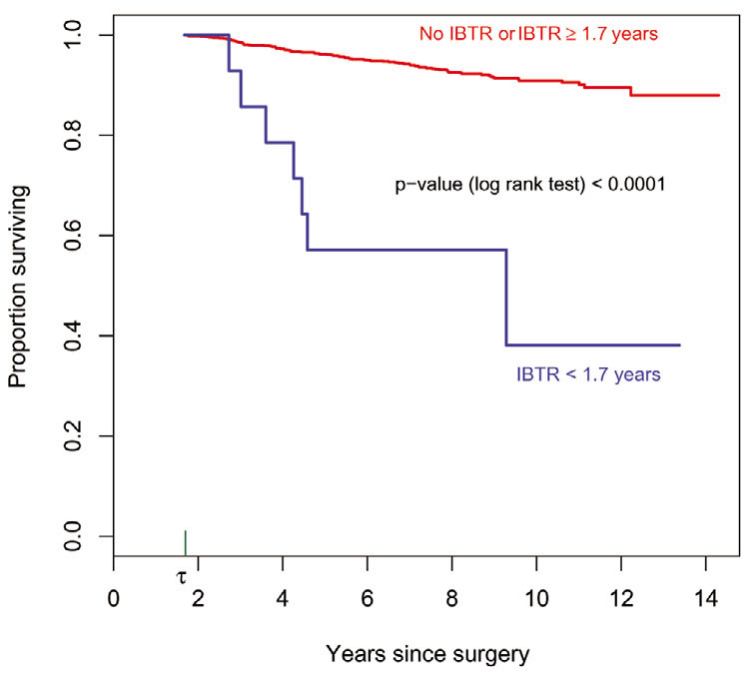
Kaplan–Meier estimate of disease-specific survival by IBTR status at 1.7 years (τ) after surgery. IBTR, ipsilateral breast tumour recurrence.

Twelve percent of the 117 women who died from or with disease during this study were diagnosed with a previous IBTR, with an overall median time to death of 4.3 years (SD 2.5). Five years after surgery, 67 of 117 deaths had occurred and the percentage surviving was 95%. This percentage decreased to 87% by 12 years from surgery. With stratification on IBTR status at the landmark time point (IBTR ≤ 1.7 years, IBTR > 1.7 years or no IBTR), Figure 3 reveals that the proportion surviving at 5 years is only 0.57 if the IBTR was diagnosed within 1.7 years from surgery (*n *= 15). In contrast to this poor survival outcome, women diagnosed with an IBTR more than 1.7 years from surgery (*n *= 83) or not diagnosed with an IBTR (*n *= 1,422) so far and alive at 1.7 years from surgery had 5-year survival rates of 0.94. At the latest time of death (7.6 years) for the early IBTR group, 38% were alive, which was significantly less (log-rank test *p *≤ 0.0001) than the 91% surviving in the non-IBTR or IBTR > 1.7 years group. Inspection of the pointwise test statistics reveals strong evidence that the group with an IBTR diagnosed within 1.7 years from surgery has a hazard rate different from that expected (χ^2 ^= 47.2), with many more observed deaths (7) than expected (0.81).

A multivariate Cox PH model for DSS (right side of Table 3) was fitted including age at diagnosis (less than 40 years) and IBTR occurrence as a time-dependent covariate, and using all factors having weak statistical significance in univariate analysis (left side of Table 3; *p *< 0.1) as well as the three treatment variables. In contrast with the analysis of IBTR-free survival, age at primary diagnosis < 40 years was not significant in the multivariate model. The diagnosis of IBTR increased the risk of death with or from breast cancer by 2.6. In this model, histological grade '2' or 3 was most strongly associated with the risk of mortality, with a RR 8.6-fold that of histological grade 1. Increasing tumour size and negative progesterone receptor status also retained significance in the multivariate model. However, the estimated RRs for the adjuvant treatment variables changed with adjustment for other factors in the multivariate model, owing to the association of treatment received with known prognostic factors [11].

Our investigation of patient age at diagnosis as a prognostic factor for IBTR and DSS found that age was an important risk factor for IBTR but not for DSS. The Kaplan–Meier survival estimates of time to IBTR suggested differences between the older (age ≥ 40 years) and younger (age < 40 years) women after 5 years of follow-up but the difference was not significant on the basis of a log-rank test statistic (*p *= 0.47). Patient age at diagnosis was statistically significant at the 5% level in a multivariable Cox PH model for IBTR, with a RR of 1.89 (95% CI = 1.00 to 3.58) but not in a univariate Cox PH model for IBTR (RR = 1.50, 95% CI = 0.83 to 2.75).

### Competing risks and model assessment

The differences between the two estimated cumulative incidence curves were extremely small, suggesting that it is not necessary to account for the competing risks when assessing time-to-IBTR and DSS (data not shown).

We also fitted proportional subdistribution hazards regression models of the final multiple variable models for time to IBTR and DSS. The estimated parameters (coefficients and their standard errors) and Wald statistic *p *values were compared with those obtained from the standard Cox PH model. Generally, results were identical to two decimal places and there were no real differences in the significance of the variables in the final models (results not shown). Thus, the effects of the competing risks could safely be ignored in this study.

**Figure 4:**
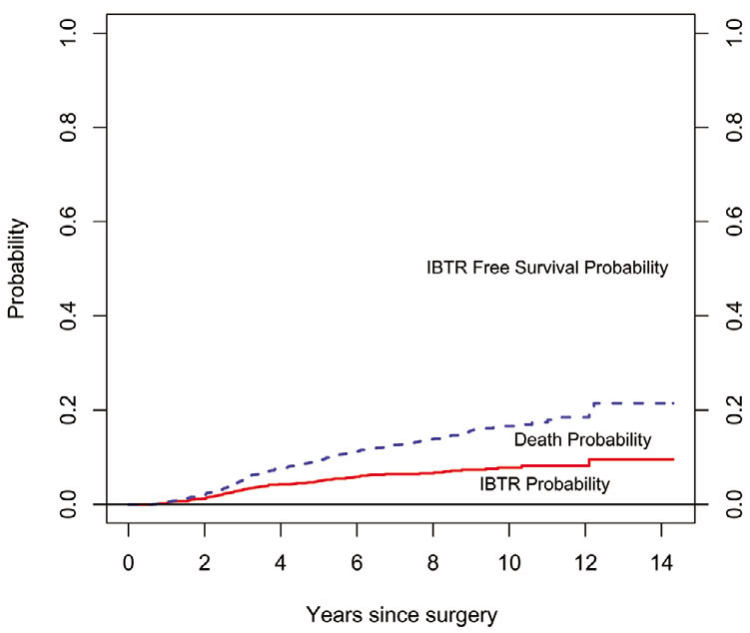
Competing risks between ipsilateral breast tumour recurrence (IBTR) and disease-specific survival.

A summary plot of the curves for the competing risks can show how changes in the probability of one event can impact the probability of the other event. In Figure 4, the IBTR cumulative incidence and the sum of the IBTR and disease-specific death cumulative incidences are plotted. At any given time, the height of the lowest curve is the IBTR probability, the distance between the two curves is the probability of dying from breast cancer, and the distance from the top of the plot (1.0) to the highest curve is the probability of being alive without IBTR. At 5 years the IBTR probability is 0.049, which is slightly higher than the probability of dying from breast cancer (0.043); the IBTR-free survival is 0.91. At the end of the study follow-up period (14.3 years) the IBTR probability is 0.095, which is now lower than the probability of dying from or with breast cancer (0.119); the IBTR-free survival is reduced to 0.78.

The global test of proportional hazards for the IBTR multivariate regression model was non-significant, as were univariate tests for non-proportionality for each prognostic factor in this model. Hence, there was no evidence that the proportional hazards assumptions were not met. For the disease-specific multivariate regression model, however, the global test of proportional hazards was significant, indicating evidence against this assumption. Two variables in this final model, oestrogen receptor status and hormone therapy, also showed evidence of non-proportionality. Plots of the scaled Schoenfeld residuals along with a fitted linear spline indicated that being negative or equivocal for oestrogen receptor status was associated with higher death rates for about the first 5 years after surgery but not thereafter. Hormone therapy also had a negative trend in these plots; the apparent benefit of this therapy developed slowly and did not appear until nearly 5 years from surgery. Thus, the estimated RRs for these time-dependent factors represent an average effect over the follow-up time (results not shown).

## Discussion

Our study adds to the literature on local recurrence after BCS in four respects. First, the incidence of local recurrence (6%) in this community setting is comparable to that seen in clinical trial data. This level was achieved despite the fact that 15% of our patients did not receive radiotherapy. Because therapy was at the discretion of the treating physician, we assume that radiotherapy was not thought necessary or suitable for these women. Second, the median time to IBTR of 3.1 years closely parallels that (46 months) seen in a similarly treated, node-negative cohort as published by Cowen and colleagues [21]. Third, in multivariate analysis, we found that young age (less than 40 years) was associated with a risk of local recurrence of 1.89 times that of older women. The only other factor to retain significance in a multivariate analysis was the presence of intraductal disease. However, some variables occurred infrequently, resulting in low power to detect an association. Finally, this observational study is consistent with the 15-year update of the National Surgical Adjuvant Breast and Bowel Project B06 trial in which age < 40, presence of an intraductal component as well as nuclear grade were independently associated with the time to IBTR [22].

The 5-year overall survival after IBTR in women treated with BCS is between 50 and 75% [21,23-27]. Our data demonstrate once again that an early local recurrence after conservative surgery is associated with poorer DSS (DSS at over 9 years from surgery is 91%, versus 38%). In this series, lymphatic invasion, large tumour size, high histological grade, negative hormone receptor status and presence of local recurrence were each associated with reduced DSS in a multivariate model. This effect has been noted by Veronesi and colleagues (failure within 2 years) [26], Whelan and colleagues (failure within 1 year) [27] and Haffty and colleagues (failure within 4 years) [23].

Young women have previously been found to have a worse prognosis than older women, in terms of increased rates of both local recurrence [28-30] and mortality [31-33]. Although close to nominal statistical significance, we did observe an increased incidence of local recurrence in women under 40 years old (9.4%). These young women had increased rates of other unfavourable prognostic factors (larger tumour size, higher tumour grade). Because only one woman in this younger age group had a local recurrence and subsequently died from or with breast cancer, it was not possible to determine whether higher rates of local recurrence contribute to their poorer survival.

The competing risk analysis revealed that, in this study, the effects of the competing risks could be ignored. The summary plot of the cumulative incidences of IBTR and death from or with breast cancer (Figure 4) showed how the probability of developing an IBTR versus dying from breast cancer changes over the time since surgery. After surgery, women have a greater likelihood of developing a local recurrence. However, as the length of time since surgery increases, the likelihood of dying from or with breast cancer exceeds the likelihood of developing a local recurrence.

## Conclusion

Our results support published data on factors associated with local recurrence, including the impact of early-onset breast cancer. We also demonstrate that the early occurrence of IBTR is associated with a relatively poor 5-year DSS of only 57%. The repeated identification of this adverse effect suggests that women with early local recurrence should be targets for clinical studies of 'pseudo-adjuvant' systemic therapy.

## Abbreviations

BCS = breast-conserving surgery; CI = confidence interval; DSS = disease-specific survival; IBTR = ipsilateral breast tumour recurrence; PH = proportional hazards; RR = relative risk.

## Competing interests

The authors declare that they have no competing interests.

## Authors' contributions

MKN and MEB conceived of the study. KAK and AT performed the statistical analyses. The manuscript was drafted by MKN and KAK. SBB provided oversight of the data analyses and critical revision of the manuscript for its intellectual content. MEB, ILA and SBB designed the original study and continue to coordinate follow-up data collection. All authors read and approved the final manuscript.

## Figures and Tables

**Table 1:** Patient, tumour and treatment characteristics

Characteristic	All eligible	Age < 40 years	Age ≥ 40 years
Number of patients	1,540	128	1,412
Number of IBTRs	98	12	86
Number of deaths (from or with disease)	117	12	105

Characteristic	Number (%)	Number (%)	Number (%)

Menopausal status			
Pre- or perimenopausal	544 (35.3)	127 (99.2)	417 (29.5)
Postmenopausal	996 (64.7)	1 (0.8)	995 (70.5)
Size of tumour			
Less than 1.0 cm	319 (20.7)	15 (11.7)	304 (21.5)
1 to 2 cm	689 (44.7)	43 (33.6)	646 (45.8)
2 to 5 cm	516 (33.5)	64 (50.0)	452 (32.0)
Greater than 5 cm	16 (1.0)	6 (4.7)	10 (0.7)
Oestrogen receptor			
Negative	242 (15.7)	46 (35.9)	196 (13.9)
Equivocal	67 (4.4)	14 (10.9)	53 (3.8)
Positive	858 (55.7)	57 (44.5)	801 (56.7)
Unknown/not done	373 (24.2)	11 (8.6)	362 (25.6)
Progesterone receptor			
Negative	335 (21.8)	47 (36.7)	288 (20.4)
Equivocal	104 (6.8)	15 (11.7)	89 (6.3)
Positive	727 (47.2)	55 (43.0)	672 (47.6)
Unknown/not done	374 (24.3)	11 (8.6)	363 (25.7)
Histological grade			
Grade 1	306 (19.9)	12 (9.4)	294 (20.8)
Grade '2'	563 (36.6)	38 (29.7)	525 (37.2)
Grade 3	428 (27.8)	62 (48.4)	366 (25.9)
Unknown/not done	243 (15.8)	16 (12.5)	227 (16.1)
Lymphatic invasion (1 unknown)			
Yes	202 (13.1)	19 (14.8)	183 (13.0)
No	1,337 (86.8)	109 (85.2)	1,228 (87.0)
Vascular invasion (2 unknown)			
Yes	55 (3.6)	6 (4.7)	49 (3.5)
No	1,483 (96.3)	122 (95.3)	1,361 (96.4)
Intraductal component (2 unknown)			
Present	957 (62.2)	69 (53.9)	888 (62.9)
Absent	581 (37.7)	59 (46.1)	522 (37.0)
Multicentricity			
Yes	95 (6.2)	8 (6.3)	87 (6.2)
No	1,437 (93.3)	119 (93.0)	1,318 (93.3)
Unknown/not done	8 (0.5)	1 (0.8)	7 (0.5)
Radiation therapy	1,300 (84.4)	118 (92.2)	1,182 (83.7)
Adjuvant treatment			
Hormone therapy	758 (49.2)	23 (18.0)	735 (52.1)
Chemotherapy	239 (15.5)	55 (43.0)	184 (13.0)
Hormone and chemotherapy	55 (3.6)	5 (0.3)	50 (3.2)

IBTR, ipsilateral breast tumour recurrence.

**Table 2:** Results of IBTR-free survival analysis by univariate and multivariate Cox proportional hazards models

Prognostic variable	Univariate			Multivariate		
	RR	95% CI	*p*	RR	95% CI	*P*
Age at primary diagnosis^a^	1.50	0.83–2.75	0.18	1.89	1.00–3.58	0.05
Intraductal (yes versus else)	1.69	1.09–2.64	0.02	1.81	1.15–2.85	0.01
Multicentricity (yes versus else)	2.10	1.15–3.84	0.02	1.54	0.83–2.85	0.17
Histological grade						
'G2' & GG3 versus G1	1.39	0.76–2.53	0.29	1.59	0.87–2.94	0.13
Not done and don't know versus G1	2.50	1.29–4.85	0.01	2.40	1.23–4.67	0.01
Tumour size						
1–2 cm versus <1 cm	1.12	0.70–1.80	0.64			
>2 cm versus <1 cm	1.18	0.69–2.01	0.55			
Oestrogen receptor status^b^	0.87	0.52–1.47	0.61			
Progesterone receptor status^b^	1.46	0.97–2.21	0.07			
Menopausal status^c^	1.12	0.74–1.70	0.58			
Vascular invasion (yes versus else)	0.59	0.15–2.39	0.46			
Lymphatic invasion (yes versus else)	0.97	0.53–1.78	0.48			
Radiation therapy versus none	0.30	0.20–0.45	<0.000001	0.29	0.19–0.44	<0.000001
Adjuvant treatment						
Hormone therapy versus none	0.62	0.41–0.93	0.02	0.56	0.36–0.85	0.01
Chemotherapy versus none	0.47	0.22–1.02	0.06	0.45	0.20–1.02	0.06

98 IBTRs among 1,540 eligible women. IBTR, ipsilateral breast tumour recurrence; CI, confidence interval; RR, relative risk. ^a^Age < 40 years versus age ≥ 40 years; ^b^negative and equivocal versus positive and don't know and not done; ^c^premenopausal and perimenopausal aged < 50 years versus postmenopausal and perimenopausal aged ≥ 50 years.

**Table 3:** Results of disease-specific survival analysis by univariate and multivariate Cox proportional hazards models

Prognostic variable	Univariate			Multivariate		
	RR	95% CI	*p*	RR	95% CI	*p*
Previous IBTR (yes versus else)	3.04	1.72–5.35	0.0001	2.58	1.41–4.72	0.002
Age at primary diagnosis^a^	1.17	0.64–2.13	0.61	0.79	0.42–1.46	0.45
Intraductal (yes versus else)	0.90	0.62–1.29	0.56			
Multicentricity (yes versus else)	1.01	0.49–2.08	0.97			
Histological grade						
'G2' and G3 versus G1	14.64	3.64–58.9	0.006	8.59	2.09–35.36	0.003
Not done and don't know versus G1	7.86	1.81–34.2	0.0002	4.73	1.07–20.94	0.04
Tumour size						
1–2 cm versus <1 cm	2.74	1.49–5.03	0.001	1.95	1.05–3.64	0.04
>2 cm versus <1 cm	4.97	2.71–9.12	< 0.00001	2.94	1.56–5.56	0.0009
Oestrogen receptor status^b^	2.77	1.91–4.01	< 0.00001	1.23	0.75–2.04	0.41
Progesterone receptor status^b^	3.56	2.47–5.14	< 0.00001	2.15	1.36–3.39	0.001
Menopausal status^c^	1.18	0.81–1.72	0.38			
Vascular invasion (yes versus else)	1.78	0.83–3.83	0.14			
Lymphatic invasion (yes versus else)	2.35	1.55–3.57	0.00006	1.78	1.17–2.72	0.008
Radiation therapy versus none	1.19	0.70–2.01	0.53	1.18	0.67–2.09	0.57
Adjuvant treatment						
Hormone therapy versus none	0.66	0.46–0.97	0.03	0.75	0.50–1.15	0.19
Chemotherapy versus none	1.47	0.92–2.36	0.11	0.62	0.36–1.05	0.07

117 deaths from or with disease among 1,540 eligible women. CI, confidence interval; IBTR, ipsilateral breast tumour recurrence; RR, relative risk. ^a^Age < 40 years versus age ≥ 40 years; ^b^negative and equivocal versus positive and don't know and not done; ^c^premenopausal and perimenopausal aged < 50 years versus postmenopausal and perimenopausal aged ≥ 50 years.
